# Acceptability of HPV screening among HIV-infected women attending an HIV-dedicated clinic in Abidjan, Côte d’Ivoire

**DOI:** 10.1186/s12905-020-01021-6

**Published:** 2020-07-28

**Authors:** Keitly Mensah, Nelly Assoumou, Véronique Duchesne, Dolorès Pourette, Pierre DeBeaudrap, Alexandre Dumont

**Affiliations:** 1 Institut Recherche et Développement, Université de Paris, Centre Population et Développement (CEPED), 45 Rue des Saints-Pères, 75006 Paris, France; 2 Programme PAC-CI, Site ANRS de Côte d’Ivoire, Abidjan, Côte d’Ivoire

**Keywords:** Cervical cancer, HIV, Health belief model, Self-sampling, Screening, Qualitative, Côte d’Ivoire, Sub-Saharan countries, Women’s health

## Abstract

**Background:**

Cervical cancer incidence is high among women living with HIV due to high-risk HPV persistence in the cervix. In low-income countries, cervical cancer screening is based on visual inspection with acetic acid. Implementing human papilloma virus (HPV) screening through self-sampling could increase women’s participation and screening performance. Our study aims to assess the preintervention acceptability of HPV screening among HIV-infected women in Abidjan, Côte d’Ivoire.

**Methods:**

Applying the Health Belief Model theoretical framework, we collected qualitative data through in-depth interviews with 21 HIV-infected women treated in an HIV-dedicated clinic. Maximum variation sampling was used to achieve a diverse sample of women in terms of level of health literacy. Interviews were recorded and transcribed with the participants’ consent. Data analysis was performed using NVivo 12.

**Results:**

Screening acceptability relies on cervical cancer representations among women. Barriers were the fear of diagnosis and the associated stigma disregard for HIV-associated health conditions, poor knowledge of screening and insufficient resources for treatment. Fees removal, higher levels of knowledge about cervical cancer and of the role of HIV status in cancer were found to facilitate screening. Healthcare providers are obstacle removers by their trusting relationship with women and help navigating through the healthcare system. Self-confidence in self-sampling is low.

**Conclusions:**

Free access to cervical screening, communication strategies increasing cervical cancer knowledge and healthcare provider involvement will foster HPV screening. Knowledge gathered through this research is crucial for designing adequate HPV-based screening interventions for women living with HIV in this setting.

## Background

Cervical cancer is a leading cause of cancer-related death in countries in sub-Saharan Africa (SSA) [1]. The disease is due to the persistence of high-risk oncogenic human papilloma virus (HPV) in the cervix [1]. Cervical cancer incidence is high among women living with HIV due to a higher frequency of HPV infection and an impaired HPV clearance, responsible for longer persistence of HPV [2, 3]. Screening of precancerous lesions and HPV immunization are efficient prevention strategies to reduce the cervical cancer burden [4–6]. While HPV immunization is not available worldwide [7], early screening has reduced cervical cancer incidence and the related mortality rate, especially in high-income countries [8–10]. In SSA, cervical cancer prevention relies on visual inspection with acetic acid (VIA), which is considered the most cost-effective strategy [11–13]. Because VIA has limitations [14–17], HPV-based screening is a promising alternative strategy [18, 19]. Moreover, self-sampling method, which avoids examination by a healthcare provider, could increase participation in cervical cancer screening [20].

In Côte d’Ivoire, cervical cancer is the leading cause of cancer-related death among women living with HIV [21], and, compared to other countries in West Africa, the HIV prevalence is high among women (4.1%) [22]. The country provides services to the HIV-infected population; as part of these services, women receive gynecological care and screening for precancerous lesions using VIA [23, 24]. In March 2019, CEPREF (Center for Care and Training), an HIV-dedicated clinic in Abidjan, Côte d’Ivoire, planned to introduce a self-sampling HPV-based screening for the prevention of cervical cancer among women living with HIV as part of the ANRS 12375 AIMA-CC study (clinicaltrial.gov: NCT03789513).

This study aims to assess the acceptability of HPV screening among HIV-infected women before the implementation of this method to adapt it to the societal context in Abidjan.

## Methods

### Setting

Participants were recruited at CEPREF, a public clinic located in a popular district in Abidjan, Cote d’Ivoire, in November 2018. The center is dedicated to the HIV-infected population and offers antiretroviral treatment, primary HIV care and more specific care, including gynecologic care. Cervical cancer screening activities using VIA were initiated in 2011, with a total of 2212 tests performed between 2015 and 2018. Of the 10,461 HIV-infected patients followed up at the clinic, 69% were women and 5654 (54%) were aged 25 to 55 years during this period. Therefore, we estimated that approximately 35% of eligible women participated in cervical screening over this period of time.

Chigata is a local association that supports isolated women, mothers with their children and orphans living with HIV. This association is known in the district and works closely with CEPREF. Additionally, it offers to its members regular training provided by healthcare professionals from the University Hospital. Training topics cover HIV (diagnosis, prevention, and treatment) and HIV-related diseases.

### Study design

We conducted a preintervention qualitative study based on semidirected interviews with women considered as targets for cervical cancer screening. The semistructured qualitative guide covered the following topics: knowledge about women’s health issues in general and the effect of HIV on women’s health; knowledge, beliefs and experiences with respect to cervical cancer and cervical cancer screening; and opinions about the use of self-sampling and HPV testing as a new screening strategy. The interview guide developed for this study is provided as Additional file 1.

### Participants and recruitment

Eligible women were 25- to 55-year-old HIV-infected women treated at CEPREF and French speakers. Our sample was equally divided between women who were members of Chigata association (group A) and those who were not members (group R) to assess how acceptability may vary between the general population and women involved in an association.

The women in group R were recruited by midwives and nurses from among patients attending the CEPREF center between November 12 and November 23, 2018. They were invited to be interviewed while waiting for their medical visit. Women of group A were contacted by phone by the head of Chigata during the same period and agreed to meet us for an individual interview at a specific time at the CEPREF center.

### Data collection

Interviews were scheduled at a convenient time and were conducted in a neutral meeting room of the clinic. Participants received an informational note explaining the study, agreed to participate and signed a written consent form. Interviews were conducted in French by two female researchers either together or on a one-to-one basis. The interviews lasted between 30 and 60 min and were conducted using the semidirected interview guide. Reimbursement of transportation fees was provided at the end of the interview. The study received ethical approval from the Ethics Committee of Côte d’Ivoire as part of the AIMA-CC study.

### Analysis

All interviews were digitally recorded and transcribed before being coded using NVivo 12 software. Inductive content analysis was employed to analyze and interpret the qualitative data. Emerging themes were grouped according to an analysis framework based on the Health Belief Model (HBM).

The HBM has been extensively used in health promotion to investigate health-related behaviors [25] and, more specifically, to explain and predict cervical cancer screening behaviors among HIV-infected and uninfected women [26]. It is based on the premise that health-related behaviors are driven by six factors: perceived severity, perceived susceptibility, perceived barriers, perceived benefits, cues to action and self-efficacy (Table 1). This framework was used to analyze and provide an in-depth understanding of various factors that drive the acceptability of HPV screening at the preimplementation stage.

**Table 1:** HBM dimensions and their definitions

Dimension	Definition
**Perceived severity**	Refers to women’s views on the seriousness of contracting a disease, here, cervical cancer.
**Perceived susceptibility**	Refers to women’s perceptions of the risk of acquiring the disease.
**Perceived barriers**	Refers to women’s feelings on the obstacles to performing a behavior, here, being screened for cervical cancer.
**Perceived benefits**	Refers to women’s perceptions of the effectiveness of this screening to reduce the threat of cervical cancer.
**Cues to action**	This is the stimulus needed to trigger the decision-making process to accept a recommended health action.
**Self-efficacy**	Refers to the level of women’s confidence in their ability to successfully perform the screening. It usually refers to knowing the screening location and feeling confident to achieve the different screening steps.

## Results

We performed 21 in-depth interviews with 12 women attending the clinic (group R) and 9 members of Chigata (group A). Participants’ characteristics are presented in Table 2. All women from group A already had a previous VIA screening, and most of them had been diagnosed with HIV infection for more than 10 years. There were more variations in screening status and length of time since HIV diagnosis among women of group R.

**Table 2:** Study population characteristics

Variable (N)	Group A Association member (*n* = 9)	Group R Nonmember (*n* = 12)
**Age**		
25–30	0	3
31–40	1	6
41–50	3	3
> 50	5	0
**Education level**		
None	0	4
Elementary school	3	3
Middle school	3	4
High school	2	1
College or professional degree	1	0
**Marital status**		
Single	2	2
Divorced, separated	1	0
Married	2	4
Not married, cohabiting	2	3
In a relationship	0	3
Widow	2	1
**Length of time since HIV diagnosis**		
< 1 year	0	2
1–5 years	0	3
5–10 years	2	2
> 10 years	7	3
NA	0	2
**Previously underwent a VIA screening**		
Yes	9	2
No	0	10

The main findings of qualitative analysis presented below are summarized in Table 3.

**Table 3:** Themes generated using the HBM framework

**Perceived severity**	▪ Cervical cancer is a deadly disease	▪ Cervical cancer is perceived as an additional burden after HIV	
**Perceived susceptibility**	▪ Existing link between womanhood and vulnerability to cancer	▪ Existing link between HIV infection and cervical cancer	▪ Existing link between sexual intercourse and cervical cancer
**Perceived barriers**	▪ Fear of an invisible disease	▪ Fear of a diagnosis perceived as an additional burden with associated stigma	▪ Lack of financial resources ▪ Lack of knowledge
**Perceived benefits**	▪ Maintaining health	▪ Early stage treatment availability	
**Cues to action**	▪ Knowledge of cervical cancer and awareness of treatment	▪ Reliance on and trust in healthcare workers	▪ Free procedure
**Self-efficacy**	▪ Lack of confidence in performing self-sampling	▪ Health system navigation using healthcare workers support	▪ Uptake in confidence after first screening experience

### Perceived severity

All the women interviewed knew the word “cancer”. They had heard about cervical cancer through TV campaigns, educational sessions at CEPREF or specific training. For all, cervical cancer was a serious condition associated with death: “*But cancer, if its treatment doesn’t exist, you’d rather die” (> 50 y, group A, HIV for > 10 y, screened).* The majority of women knew or heard of someone who died from cancer (primarily breast cancer). In both groups, some women considered cancer incurable: *“This close friend, despite all the treatment she got, she died.” (> 50 y, group A, HIV for > 15 y, screened). Some?* Women from group R had heard that the disease can be treated at an early stage; however, they were doubtful: *“They say that if you’re screened early you can be cured. [ … ] Maybe if it’s detected early you can be cured … I don’t know.” (31–40 y, group R, HIV for 16 y, not screened).* All the women attributed their fear of the disease to its invisibility and their inability to see early symptoms.

During the interviews, all women expressed that HIV was a burden that was already difficult to cope with. Most women referred to cancer as an additional burden, worse than HIV. One statement captured the general feeling: *“You can’t say that one disease is better than the other, but AIDS is better. [ …] because with AIDS, if you take your pills correctly it can extend your life while for the cancer it’s not that easy.” (41–50 y, group R, HIV for 8 y, not screened).*

### Perceived susceptibility

Most women perceived womanhood as making them more vulnerable to infections. When asked about the potential relationship between HIV and cancer, women from group R did not associate their HIV status with a higher risk of cancer. In contrast, women from group A were aware of the potential effect of HIV on cancer development: *“You, you’re living, and the virus is spreading. So with the cancer, you will be affected. But the other woman, as she doesn’t have the virus in her body, to get affected it will be harder”(> 50 y, group A, HIV for > 15 y, screened).*

All the women considered sexual activity as the transmission path of HPV; therefore, women without sexual encounters did not feel concerned by the screening: *“Why would I get screened? I don’t have any man, I’m alone … [ …] I don’t have any sexual encounters, nothing.” (41–50 y, group R, HIV for > 15 y, screened)*.

### Perceived barriers

Fear of a diagnosis was expressed as the main reason to avoid the screening procedure. In addition, all women who had a screening history expressed their fear of a diagnosis while waiting for the result. In both groups, living with HIV enhanced this fear, as women were already dealing with their HIV status and treatment: *“We’re already living with a problem that we haven’t solved yet. [ …] When you see that you’ve already got a problem and this disease people are talking about is not a small disease, it’s a reason to be scared.” (41–50 y, group A, HIV for > 10 y, screened).* Some women emphasized the consequences of a diagnosis and feared being isolated: *“If people know, I’ll be in danger and my family as well. Nobody will come close to me.”* (*41–40 y, group R, HIV for > 10 y, not screened*). A general finding was that most women, due to the stigma of HIV, had little support and that issues such as cancer were not discussed in their personal environment.

Lack of financial resources prevented women from starting the screening process. When asked what their reaction to a positive screening would be, many women raised this issue. Money was also a barrier to an annual screening among women who knew that they should be screened every year: *“You don’t have any money, how do you do? I used to do it, it was free. [ …] if I hear that I don’t have to pay, I’ll come back.” (> 50 y, group A, HIV for > 15 y, screening).*

Lack of knowledge appeared as a barrier, as few women had specific knowledge about cervical cancer. In group R, only two women could cite some cervical cancer symptoms. *“[Healthcare workers during information sessions] said that, often, when you have a sexual encounter with a guy, you bleed, or that when you want to do your intimate cleaning, you see that you’re bleeding.” (31–40 y, group R, HIV diagnosis date unknown, not screened)*.

### Perceived benefits

Women had previously heard about cancer through either health professionals or national media. Most women, even those who had not been previously screened, mentioned the need for screening. *“Well, we are asked to get screened to know. Because you can live, have it without knowing it!” (41–50 y, group R, HIV for 8 y, not screened).*

Additionally, most of these women were aware of the curable aspect of cervical cancer: “*By listening and asking for advice, I know that cervical cancer is treatable. [ …] Seriously I was relieved.” (> 50 y, group A, HIV for > 15 y, screened).*

Women who already underwent a VIA screening did not associate it with discomfort. Moreover, they exhibited health awareness: *“I’m protecting myself from many things. I’m telling myself that by doing it, there are diseases that I’ll avoid or even death. Because I don’t want to die from a disease” (41–40 y, group R, HIV for > 10 y, not screened).* Women from group A better articulated the need to stay healthy: *“I should make a plan and take some time from my personal agenda to have some time to take care of myself.” (41–50 y, group A, HIV for 10 y, screened).*

### Cues to action

Healthcare workers were quoted as a resource to help women overcome their fear of the procedure and the consequences of the diagnosis by delivering information about cervical cancer and screening. Women from both groups mentioned that the time spent discussing this issue and the support they received from healthcare workers gave them the courage to get screened: *“Yes … I listened to them [the midwives], I was a little bit scared but the way they spoke, it motivated me to do it [the screening] for myself” (41–50 y, group R, HIV for > 15 y, screened)*.

Another important facilitating factor for all women was that the procedure was free. A woman with no screening history compared cervical cancer screening to HIV care: *“We should not have to pay. It has to be free, the way people in the AIDS/HIV field are taking care of us, you have to take care of us the same way.” (41–40 y, group R, HIV for < 1 y, not screened).* Women who had previously been screened stated that gratuity played a major role in their decision to get screened: *“The free access also encouraged me. Because when you arrive and people tell you that you have to pay, you hesitate a little bit. The free access encourages. It is the reason why when I went back home, I told women from my neighborhood to go get screened.” (41–50 y, group A, HIV for > 10 y, screened)*. Hence, free access seems to help women overcome their fear of a diagnosis and its consequences: *“Last year they said to all women that the cervical screening was free. To go and see, to not be afraid. [ …] Me, I took my courage and I went to be screened.” (> 50 y, group A, HIV for > 15 y, screened).*

The literacy level was associated with the cervical screening history. In group A, all women underwent screening once. They had a higher level of literacy about cervical cancer and more accurate knowledge of the natural disease history than in group R. *“Yes, it’s a virus, the papilloma virus. [The healthcare workers] said that you could get it at a young age and live with it for a long time then when you get older it grows.” (41–50 y, group A, HIV for > 10 y, screened).*

### Self-efficacy

When asked about location to get screened or the procedure to follow in case of a positive screening, none of the women knew the different steps. However, they all said they would rely on the healthcare workers, using them to navigate through the healthcare system.

Having already been through the whole screening procedure and the postscreening counselling increased the confidence that women had in the screening. This self-efficacy uptake was associated with the knowledge about cervical cancer gained through the process: “*It was easier the second time, as the first time they said that bleeding was the sign. As I was not having that issue, I was confident when I went.” (41–50 y, group A, HIV for > 10 y, screen).* Women’s confidence in their “good” behavior also plays a role in their self-efficacy: “*Once the screening is done when you get your results, you’re told, here you are, you have to keep behaving the good way, keep taking care of yourself. And well, as I was following it, I was not as anxious as the first time.” (> 50 y, group A, HIV for > 10 y, screened).*

Even when they considered that self-sampling was a good option, the majority of women did not trust themselves to perform it and would rather rely on healthcare workers for screening: *“[The midwife] is a specialist, so I’d rather have her doing it. I know nothing.*” *(> 50 y, group A, HIV for 10 y, screened).*

## Discussion

Our study provides useful information to adapt a self-sampling HPV-based cervical cancer screening strategy among HIV-infected women in Cote d’Ivoire. The main barriers are the unwillingness to consider an additional burden, financial issues and fears associated with cervical cancer. The main facilitators were fee removal for screening, the quality of the relationship with healthcare workers and knowledge about the disease and its prevention, highlighting the importance of addressing these issues in the further development of a screening program.

One major finding suggests that healthcare workers within an HIV-dedicated care setting have a key role in overcoming cervical screening barriers. Indeed, they appeared as facilitators of cervical screening and enhancers of self-efficacy. All the women interviewed relied on them to navigate the health system, followed their recommendations and used them to strengthen their motivation to get screened. This kind of relationship was described in Cameroon [27], where HIV-infected women felt loyalty to “their” healthcare workers. This configuration could be used to improve prescreening counselling, to understand the consequences of the lack of follow-up of screening and to overcome resistance to self-sampling.

However, as in our study, this fidelity sometimes leads to adverse effects, reflecting the ambiguity of the woman-healthcare worker relationship. For instance, women refused to get referred elsewhere. In our case, women expressed conflicting feelings due to their interest in the self-sampling method and their lack of confidence in performing it themselves and preferred to rely on healthcare workers. Similar results were found among women living with HIV in Kenya and Rwanda, with a discrepancy between the expressed acceptability of self-sampling and lack of self-confidence in performing it [28, 29]. In addition, the woman-healthcare worker relationship could reduce women’s free choice in health decisions, as some felt forced to undergo the screening, which is the opposite of empowerment. Studies exploring ethics in HIV care have noted similar issues, and this intervention could be an opportunity to monitor potential changes.

Fear of the disease and of the consequences of the diagnosis, especially the associated stigma, prevented women from being screened, as cancer is considered a double burden. While HIV-related stigma is known to reduce care-seeking behavior, some studies have also explored the stigma associated with HIV-related cancers and the stigma related to cervical cancer itself [30–32]. The findings were similar to those in our study, with women worrying that being labeled a cancer patient would lead to further social isolation. Interestingly, these studies revealed a fear of change in healthcare workers’ behavior – stigmatization, lack of interest in their case if it was judged fatal – that was not expressed in our study, suggesting that trust between women and caregivers may reduce this stigma [33–35]. Deepening the understanding of the relationship between women and caregivers through further studies would help to identify factors that could shape a trusting relationship and foster screening strategy implementation.

An important factor to reduce barriers to screening and create a community effect is offering free procedures. Fees removal is a known determinant of access to cervical screening in SSA [36] and is even more important for HIV-infected women already facing a high economic burden due to their condition [37]. Women expressed that cervical cancer care should, like HIV care, be free. Considering the positive effect of free access to maternal care [38], extending such free access to women’s health in general should increase early cancer screening. As universal health coverage is considered in SSA, integrating cervical cancer screening would help reduce cervical cancer-related mortality and incidence [39].

Literacy level also plays a major role in the decision to get screened [40]. The women from Chigata association benefit from multiple sources of information and regular training, which may have increased their health literacy and helped overcome the fear of cancer by raising their awareness of effective treatment. Preintervention acceptability of HPV screening appeared much higher among women in group A compared with women in group R. Women with a high level of health awareness are more likely to seek support and improve their health literacy level and health outcomes [36]. Improving health literacy on cervical cancer prevention could reduce multiple misunderstandings preventing women from participating in screening. This could be achieved using local TV and radio shows, counseling women living with HIV during HIV-dedicated clinics and providing posttest counseling for those who are screened. As effective health communication relies on a wide variety of channels, combining mass media that raise awareness with community-level communication (testimonies, social-media) would participate to change behaviors associated with cervical cancer screening [41].

Beyond participation in screening, integrating HPV-based screening within HIV care may have wider effects on sexual and reproductive health [42]. Early detection of premalignant lesions applies to breast cancer and may help to raise awareness about both conditions [43]. Likewise, HPV-positive results may lead to changes in sexual behavior [44, 45]. As women living with HIV already receive regular training on safe sex, it may increase their willingness to protect themselves from HPV transmission. However, it is also known that safe sex does not reflect only women’s willingness or level of awareness but also their power relationships with their partners [46]. Further studies will be needed to highlight potential changes in sexual health and behaviors induced by HPV-based screening introduction.

Though we reached data saturation, this study has limitations. First, data are restricted to women treated in a specialized urban clinic; therefore, acceptability among all women living with HIV cannot be inferred. All the women interviewed were receiving antiretroviral treatment, implying adherence to care. In addition, our findings reflect the perceptions of French-speaking women and not those of the general population, especially those of ethnic groups. Furthermore, desirability bias may have been introduced, as interviewers were introduced as doctors and researchers, and the women expressed their hope that they could help them. As the study site is linked to a research unit, women were eager to participate, and we might have overestimated the level of knowledge in our population.

## Conclusion

Our findings highlight the role that caregivers have at many points in the cervical screening process: from facilitating screening initiation to navigation in the health system. From a clinical point of view, these results provide needed information to shape adequate cervical cancer counselling. Caregivers working specifically with women living with HIV are used to adapt their messages to enhance adherence to treatment. Adding HPV-based screening to their routine activity will require them to target key points that may prevent women from getting screened or treated.

Additionally, these results will help researchers and healthcare professionals to adapt the HPV-based screening strategy to the specific context of an HIV clinic in Abidjan, Côte d’Ivoire. The new screening strategy will benefit from the specific structure of and pre-existing relationships within HIV-dedicated care centers. Further studies assessing the acceptability to women and healthcare workers of the new strategy during its implementation will be important, as they will help to design its potential integration into regular HIV care and evaluate changes in perceptions about cervical cancer associated with the implementation process.

## Supplementary information

**Additional file 1.** Semistructured qualitative guide for interviews with women living with HIV. Qualitative guide used during semistructred inteviews with women living with HIV.

## Data Availability

The datasets used and/or analyzed during the current study are available from the corresponding author on reasonable request.

## Abbreviations

ANRS: Agence Nationale de la Recherche sur le VIH et les Hépatites (National French Agency for Research on HIV and Hepatitis)
AIMA-CC study: AIDS Malignancies in Africa & Asia – Cervical Cancer Study
CEPREF: Centre de Prise en Charge et Formation (Center for Care and Training)
HBM: Health Belief Model
HIV: Human immunodeficiency virus
HPV: Human papilloma virus
SSA: Sud-Saharan Africa
VIA: Visual inspection with acetic acid
