# Supplementary material for: Acceptability of HPV screening among HIV-infected women attending an HIV-dedicated clinic in Abidjan, Côte d’Ivoire
Source: BMC Womens Health. 2020 Jul 28;20:155. doi: 10.1186/s12905-020-01021-6 (PMC7385896; doi:10.1186/s12905-020-01021-6)
Supplement: Supplementary file 1 — Additional file 1. Semistructured qualitative guide for interviews with women living with HIV. Qualitative guide used during semistructred inteviews with women living with HIV. [file 12905_2020_1021_MOESM1_ESM.pdf]

Additional File 1

Semi structured qualitative guide for interviews with women living with HIV

General Data

Date	Education (level of study or degree)
Location	Employment
Age	Associative or non-associative member

Open questions are asked first, if the participant do not respond spontaneously, use the more detailed question to guide them. These questions are just suggestions, they don't have to be asked every time and should be adapted to the respondent. Before moving to the following section, please ensure that all the areas have been explored through the interview.

I. Social characteristics and lifestyle

1) Living conditions

First, could you tell me a little more about yourself ?

- Where do you live? How long have you lived there? Are you a landlord or a tenant?

- Are you married? (If yes) Does your husband have other wives?

(If not married) Do you have anyone in your life (a fiancé, a boyfriend)? *(If yes)* For how long? What are your plans together (marriage, engagement, a child, living together...)?

- What kind of work do you do? How much do you earn approximately per month? How do you manage this income? Does it allow you to support yourself? Do you manage to put money aside (tontine, golden cowries, savings bank, etc.)?

If not and she can't manage to support herself: How do you support yourself (help from siblings, parents, friends...)?

If she is married: Has you husband been to school? If yes, what is his school level (last class attended)? What does your husband do for living? Do you know how much he earns?

- Have you been to school? If yes, what is your school level (last class attended)?
- Do you have children? How many? How old are they?

Can you describe a day at home? What do you do from the time you wake up until you go to bed?

II. Awareness and Perception of Cervical Cancer Screening

1) Knowledge and perceptions of cervical cancer

Do you know of any diseases that only affect women? :

- Which one? What part of the body do they affect?
- Are these diseases different in women living with HIV in comparison with women without HIV?
- What can be done to treat these diseases? What can be done to prevent them?

Can you tell me what you know about the uterus and cervical cancer?

- Have you ever heard of cervical cancer? *If yes*: Where was it, with whom, and on what occasion? What information were you given?
- What causes cervical cancer (a virus, a curse, etc.)?
- When you hear about cervical cancer, how do you feel?
- Do you think you are concerned about cervical cancer? If so, why? If not, why not?
- What do you think about it? Do you know any women who have it? *If yes:* What are your relationships with them?

How would you react if you found out you had this disease?

- Are there women who are more likely to have cervical cancer? If so, which ones?
- Do you know if this is a treatable disease? *If yes:* How can it be treated? Where should we go? *If no:* Why?

2) Knowledge and perceptions of UCC screening

What are the ways to find out whether or not you have this disease?

- *If the person is talking about screening:* How do you know about screening? Do you know where it can be done? How much do you have to pay for it? What do you know about screening? How does it work? Do you know how often screening can be done?

- What difficulties have you encountered, or do you think you will encounter in getting tested?

- What could help you to get screened?

- What does your partner think about screening?

III. Stress related to cervical cancer testing in women living with HIV

How do you feel about getting tested for cervical cancer?

- Have you ever been screened before?

- *If yes:* On what occasion have you done it? What prompted you to do it? In which facility did you do the screening? Why did you choose this facility? Who took care of you during the screening? How did it go? Are there professionals with whom you feel more comfortable doing the screening?

- How was the announcement of the result made? How long did it take to get the results? How did you experience the waiting time?

- *If never tested, while aware of the screening:* Why did you not want to do the screening (fear of the result, cost, distance...)? Do you plan to do it one day? Under what conditions would you do it?

IV. Perception of self-sampling

Let me show you something. Show a self-sampling diagram and explain the technique.

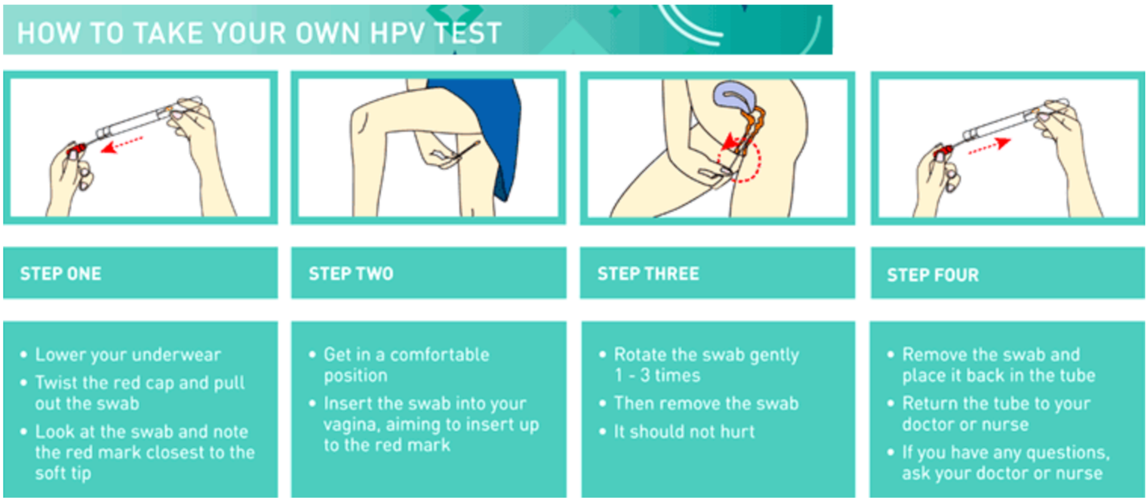

This image is adapted from Garrow SC et al. The diagnosis of chlamydia, gonorrhoea, and trichomonas infections by self-obtained low vaginal swabs in remote northern Australian clinical practice. Sex Transm infect. 2002 Aug. 78 (4) 278-81.

Figure from Self-collection of HPV samples: A guide for GPs by Dr Dr Lara Roeske (https://www1.racgp.org.au/newsgp/gp-opinion/self-collection-of-hpv-samples-a-guide-for-gps)

What do you think of this technique?

- Would you feel comfortable doing that? *If not:* why not?
